# The Age-Related Risk of Severe Outcomes Due to COVID-19 Infection: A Rapid Review, Meta-Analysis, and Meta-Regression

**DOI:** 10.3390/ijerph17165974

**Published:** 2020-08-17

**Authors:** Karla Romero Starke, Gabriela Petereit-Haack, Melanie Schubert, Daniel Kämpf, Alexandra Schliebner, Janice Hegewald, Andreas Seidler

**Affiliations:** 1 Institute and Policlinic of Occupational and Social Medicine (IPAS), Faculty of Medicine Carl Gustav Carus, Technische Universität Dresden, 01307 Dresden, Germany; melanie.schubert@tu-dresden.de (M.S.); Daniel.Kaempf@mailbox.tu-dresden.de (D.K.); alexandra.schliebner@tu-dresden.de (A.S.); Janice.Hegewald@mailbox.tu-dresden.de (J.H.); andreas.seidler@mailbox.tu-dresden.de (A.S.); 2 Division of Occupational Health, Department of Occupational Safety and Environment, Regional Government of South Hesse, 65197 Wiesbaden, Germany; gabriela.petereit-haack@rpda.hessen.de

**Keywords:** COVID-19, age, disease severity, mortality

## Abstract

Increased age appears to be a strong risk factor for COVID-19 severe outcomes. However, studies do not sufficiently consider the age-dependency of other important factors influencing the course of disease. The aim of this review was to quantify the isolated effect of age on severe COVID-19 outcomes. We searched Pubmed to find relevant studies published in 2020. Two independent reviewers evaluated them using predefined inclusion and exclusion criteria. We extracted the results and assessed seven domains of bias for each study. After adjusting for important age-related risk factors, the isolated effect of age was estimated using meta-regression. Twelve studies met our inclusion criteria: four studies for COVID-19 disease severity, seven for mortality, and one for admission to ICU. The crude effect of age (5.2% and 13.4% higher risk of disease severity and death per age year, respectively) substantially decreased when adjusting for important age-dependent risk factors (diabetes, hypertension, coronary heart disease/cerebrovascular disease, compromised immunity, previous respiratory disease, renal disease). Adjusting for all six comorbidities indicates a 2.7% risk increase for disease severity (two studies), and no additional risk of death per year of age (five studies). The indication of a rather weak influence of age on COVID-19 disease severity after adjustment for important age-dependent risk factors should be taken in consideration when implementing age-related preventative measures (e.g., age-dependent work restrictions).

## 1. Introduction

The first cluster of cases of Coronavirus disease 2019 (COVID-19) was reported in Wuhan (Huban province, China) in December 2019. Only a little more than two months later, on March 12, 2020 the World Health Organization (WHO) announced the COVID-19 outbreak to be pandemic. Due to the high number of increasing cases and deaths, research identifying the risk factors for COVID-19 disease severity is being published at a high rate.

An increase in COVID-19 disease severity with increased patient age has been widely noted [1,2,3,4]. National health institutions such as the Robert Koch Institute (RKI) in Germany and the Centers for Disease Control (CDC) in the United States routinely report COVID-19 cases and deaths stratified by age. They have described an increase in mortality with increasing age. In its COVID-19 informational report, the RKI states “the risk of severe diseases increases steadily from 50 to 60 years of age” [5].

Due to these observations, several governments have recommended older workers, mostly starting at the age of 60, to abstain from going into the workplace during the pandemic because of the increased risk of complications due to COVID-19. After the lockdown and in the course of the relaxation of restrictions, the Federal State of Lower Saxony in Germany stated that because teachers over the age of 60 (together with persons with chronic diseases) are considered a risk group, they may be able to work from home after the presentation of a medical certificate [6]. In general, the classification of people over 60 years of age as risk persons can significantly reduce the chances of older unemployed people finding a job or make them “targets” for layoffs.

There is a major drawback in relying on the age-stratified data of disease severity for the individual assessment of the risk of a severe outcome of a disease: considering only the age-dependency of the disease course could result in a distorted picture. Other risk factors, such as cardiovascular diseases, respiratory diseases, and conditions that result in a weakened immunity, also increase with increasing age, and must be taken into account to uncover the isolated effect of age. The largest report on risk factors for death due to COVID-19 so far is based on 44,672 confirmed cases from the CDC in China [7]. Along with a case fatality rate (CFR) of 2.6% in people older than 59 years, a 6% CFR was reported for patients with hypertension, 7.3% for patients with diabetes, 10.5% for patients with cardiovascular diseases, 6.3% for patients with chronic respiratory diseases, and 5.6% for cancer patients. In addition, there is increased evidence of an increased risk of negative COVID-19 outcomes with obesity [8,9]. A recent review names smoking as a likely risk factor for adverse COVID-19 outcomes [10].

It is therefore of utmost importance to quantify the isolated effect of age in the risk for COVID-19 disease severity. The aim of this study was to do exactly this. For this purpose, we performed a systematic rapid review of the studies investigating age and COVID-19 adverse outcomes.

## 2. Methods

### 2.1. Search, Selection, and Data Extraction

On 15 May 2020, we searched Pubmed using the search strings “Covid-19” and “age”. We applied no language, region, or time restrictions. Articles in Chinese were translated automatically with the help of Google Translate. Following the Population, Exposure, Comparator, Outcome, Study Design (PECOS) scheme for the eligibility criteria of the studies (Table 1), we considered cross-sectional, case-control, and cohort studies on the general population infected with COVID-19. We defined the outcome of interest, “disease severity due to COVID-19”, as hospitalization, admission to an intensive care unit (ICU), intubation, or death due to COVID-19. Persons from the same study population with non-severe COVID-19 could be used as a comparison group, and the definition of non-severity could depend on the definition of the outcome considered. We only considered studies which estimated the adjusted risk of age on disease severity, meaning that studies which only reported age-stratified results or univariate analysis were excluded.

The resulting titles and abstracts were screened by two independent scientists to disqualify the studies which were unrelated to the defined research question. In case of disagreement on inclusion, a consensus decision was sought between the two scientists. If there was still no agreement, the decision was made by a third reviewer. The full texts of the remaining studies were again independently examined by two reviewers to determine if the inclusion criteria for this specific review were met. Likewise, in case of disagreement, a consensus decision was sought between the two reviewers, and in case of no consensus, the decision was made by third reviewer. Additionally, a manual search was performed to find additional relevant articles by screening the reference lists of key articles.

The data extraction was done by one reviewer. We tried to obtain the missing or unclear information through personal communication with the authors. The data extraction form included information on the first author and publication year, country of origin, study population, outcome, confounding, and study results.

### 2.2. Risk of Bias Assessment

For each included study, we evaluated the overall risk of bias as “low”, “high”, or “unclear”. The overall risk of bias was based on seven domains of bias, following the example used by Ijaz and colleagues [11], and considering the criteria described by SIGN (Scottish Intercollegiate Guidelines, 2012 [12] and CASP (Critical Appraisal Skills Programme [13]):

#### 2.2.1. Recruitment Procedure and Follow-Up

A low-risk study should have avoided selection bias by ensuring an adequate recruitment method, such as randomized sampling. The response rate should be 50% or more, and if not achieved, a non-participation analysis should be performed. For cohort studies, if the loss to follow-up was below 20% and there was no substantial difference between the comparison groups, the risk of bias for this domain was rated as low. Similarly, for a case-control study to be rated as having a low risk of bias for this section, both cases and control subjects should have had a response of 50% or more, and if this number was not achieved, the substantial differential selection of cases and controls should have been excluded by a non-participation analysis.

#### 2.2.2. Exposure Definition and Measurement

If the exposure (age) was accurately measured and finely categorized (i.e., in per year categories), the domain for the study was considered to have a low risk of bias. If the age was measured in large categories, such as ≥65 years vs. <65 years, the domain was considered as having a high risk of bias.

#### 2.2.3. Outcome Source and Validation

If the outcome was objectively measured to minimize bias, such as through hospital or medical records and the assessment was similar for the comparison groups, the domain was considered as having a low risk of bias. 

#### 2.2.4. Age-Dependent Risk Factors

The following major age-dependent factors (which might at least partly mediate the influence of age on the course of disease) should have been considered for this domain to have a low risk of bias: (1) diabetes, (2) hypertension, (3) coronary heart disease/cerebrovascular disease, (4) compromised immunity/cancer, (5) previous respiratory disease, and (6) renal disease. Other known risk factors of disease severity with no or little age-dependency (sex, obesity, smoking) were not considered as major age-dependent risk factors.

#### 2.2.5. Analysis Methods Including Chronology

If the adequate statistical models were used to reduce bias and control for confounding, this domain was considered as having a low risk of bias. If the risk factors were included in the model which reflected COVID-19 infection (in other words, an over-adjustment on the model), this domain was regarded as having a high risk of bias.

#### 2.2.6. Funding

This was assessed in two areas: the sources of funding and the involvement of the funding body in the research. If a study was funded by non-profit organization(s) and it was not affected by sponsors, the domain was rated as having a low risk of bias. If the sponsoring organization participated in the data analysis or the study was probably affected by the sponsors, the domain was considered as having a high risk of bias.

#### 2.2.7. Conflict of Interest

If the authors reported not having a conflict of interest, the domain was rated as having a low risk of bias. If one author had a conflict of interest, the domain was considered as having a high risk of bias.

#### 2.2.8. Overall Assessment of Risk of Bias

From the seven domains described, we considered the domains described in Section 2.2.1–Section 2.2.5 as major domains for a risk of bias, while Section 2.2.6 and Section 2.2.7 were minor domains. A “high risk” or “unclear risk” rating in any of the major domains would result in an overall “high risk of bias” assessment for each study.

When studies used either the same population or a subset of another study’s population and investigated the same outcomes, we chose the study with more precise age categories (i.e., per year of age) for the risk of bias assessment and for the subsequent meta-analysis.

### 2.3. Statistical Analysis

We used Stata version 14.2 [14] for all statistical analyses.

To obtain a precise pooled age-related risk, we decided a priori to conduct a meta-analysis if at least two studies were present with similar outcomes, exposures, and evaluating risk per year of age. We assessed statistical heterogeneity with the I^2^ statistic and assessed publication bias by observing the funnel plot asymmetry and by performing Egger’s test (*metabias*).

Since we deem adjustment for age-dependent risk factors essential for determining the isolated risk of age on disease severity, we performed a meta-regression (*metareg*) to obtain the effect of the number of important age-dependent risk factors (0–6) used in the study models (diabetes, hypertension/cardiovascular disease, compromised immunity, respiratory disease) on the relative risk. In a sensitivity analysis, we included a bivariate factor (0 = no, 1 = yes) in the model, representing whether there was an over-adjustment in the model—meaning that the model included variables which already reflected a possible COVID-19 infection (e.g., fever, dyspnea, neutrophil to lymphocyte ratio, c-reactive protein). 

## 3. Results

### 3.1. Search Results

**Figure 1:**
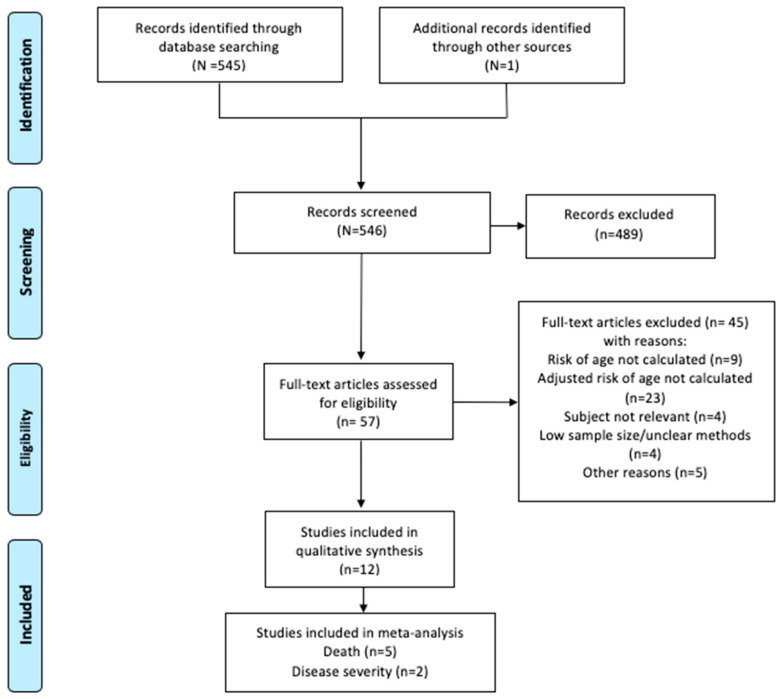
Study selection process.

Through the database and manual search, 546 studies identified were screened, resulting in 57 full-text articles assessed for eligibility (Figure 1). From these, 45 articles were excluded for the following main reasons: the risk of age was not calculated, only the crude (unadjusted) risk of age was reported, irrelevant subject, unclear methodology, and unclear outcome definition. Finally, twelve studies met our inclusion criteria. 

The study characteristics are summarized in Table 2, Table 3, Table 4 and Table 5 and below. All studies originated in China and were retrospective cohort studies, using hospitalized patients as the study population. Eight studies categorized and analyzed the patients’ age by year [15,16,17,18,19,20,21,22], three studies constructed larger age categories for their analyses [23,24,25], and one study did both [26].

Seven studies evaluated the risk of patients’ mortality [18,19,20,21,22,23,24], four evaluated the COVID-19 disease severity by building a composite index comprising of admission to the ICU, invasive ventilation, or death [15,17,25,26], and one study evaluated the admission to ICU [16]. There were studies that used the same study population or a subset of the other’s study population. An example of this is the population from Guan et al. 2020 [26] and Chen, R et al. 2020 [23], and Liang, W. [17] et al., where all three studies used the same data from 1590 participants across 575 hospitals in mainland China. Another example is that of Zhou et al. 2020 [18] and Du et al. 2020 [24]. In this case, both studies used data in a similar timeframe from a subset of hospitals included in the previous large study by Guan and colleagues [26], and therefore we considered these two studies [18,24] to have used a subset of the Guan et al. 2020 population [26]. 

### 3.2. Disease Severity by a Composite Index

#### 3.2.1. Description of Studies

A summary of the studies investigating disease severity by a composite index can be found in Table 3.

The largest study in China was a retrospective cohort study from Guan and colleagues [26], which included the clinical data of 1590 laboratory-confirmed hospitalized COVID-19 cases from 575 hospitals in China, representing almost a third of the certified hospitals in China for admitting patients with COVID-19 between 11 December 2019 and 31 January 2020. After adjustment for malignancy, chronic obstructive pulmonary disease (COPD), diabetes, hypertension, and smoking, every year of age increased the risk of severe outcome by 3.6% (HR = 1.036, 95% CI 1.022–1.050). Because this study did not consider all the necessary risk factors into its analysis (coronary heart disease/cerebrovascular and renal disease missing), it was considered to have a high risk of bias (Table 4). This study was included in our meta-analysis.

Liang et al. 2020 [17] investigated the above study’s [26] same population and outcome and performed a similar analysis using somewhat different confounders, including biomarkers that were reflective of COVID-19 infection. The study yielded similar results (per year of age HR = 1.03, 95% CI 1.01–1.05) as Guan et al. 2020 [15], but we preferred to use Guan et al. 2020 in our meta-analysis, since Liang et al. 2020 [17] used markers reflective of infection in their analysis.

Chen C. et al. 2020 [15] was a retrospective cohort made up of 150 patients admitted to the fever ward in Tongji Hospital. After adjustment for confounders, each year of age elevated the risk of severe disease by 1.9% (HR = 1.019; 95% CI 0.963–1.077), although the result was not statistically significant. This study did not consider all the necessary risk factors (diabetes, compromised immunity, respiratory disease missing). It also used several biomarkers reflective of COVID-19 infection in its analysis, so it was evaluated as having a high risk of bias. This study was included in our meta-analysis.

Meng et al. 2020 [25] investigated Chen C. and colleagues’ [15] study population, but used large age categories (0–59, 60–79, ≥80 years) for the analysis. They found a higher risk of death in older patients than in those younger than 60 years. Because of the broad age categories used, it was not used for the risk of bias or meta-analysis and Chen C. et al.’s [15] analysis was instead preferred.

#### 3.2.2. Risk of Bias

The reasons for the exclusion of any study from our risk of bias assessment and meta-analysis are given in Table 4. Again, we avoided having more than one study using the same population, and studies investigating the age effects per year of age were preferred to the studies using large age categories, such as <65 yrs. vs. ≥65 yrs. Therefore, Lian et al. 2020 [17] and Meng et al. 2020 [25] were excluded, and Guan et al. 2020 [26] and Chen C et al. 2020 [15] were included in the risk of bias assessment and in the meta-analysis.

**Figure 2:**
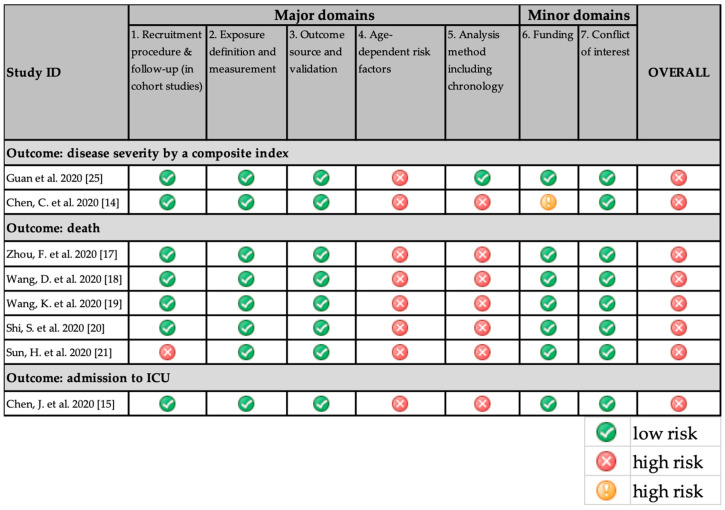
Risk of bias of studies by outcome.

Figure 2 summarizes the risk of bias (RoB) for composite endpoints of disease severity. Because all important age-dependent risk factors were not considered, both included studies [15,26] had a high risk of bias. In addition, Chen C. et al. included all the factors indicating an infection and this study was further marked down.

#### 3.2.3. Meta-Analysis and Meta-Regression

**Figure 3:**
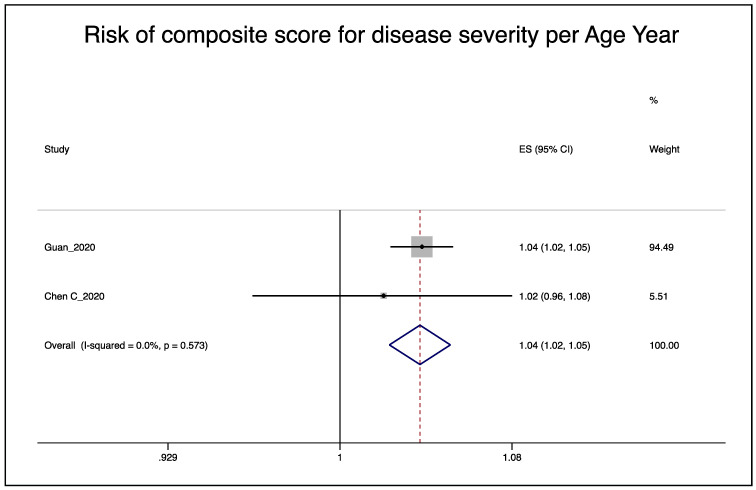
Pooled effect of the risk of age on disease severity, random-effects meta-analysis.

The pooled effect of both studies [15,26] indicates a 4% increase in the risk of severe disease per year of age (95% CI 2%–5%), Figure 3. The corresponding funnel plot indicates no evidence of publication bias (results not shown).

The meta-regression from both studies [15,26] indicated an intercept of 1.052 (95% CI 1.026–1.078) and a slope (ß) of 0.996 (95% CI 0.987–1.006). An estimate of the age-related relative risk by the number of important risk factors considered in the adjustment models can be found in Table 6. The unadjusted age effect was a 5.2% increase in disease severity per age year. The maximum number of important risk factors adjusted for by the studies was five, which resulted in a 3.5% increase in disease severity per age year.

Because there were only two studies, a sensitivity analysis of the effect of both the important risk factors and the inclusion “over-adjustment” variables in the multivariate model could not be done.

### 3.3. Death

#### 3.3.1. Description of Studies

A summary of the studies investigating death due to COVID-19 can be found in Table 4.

Chen R et al. [23] used the same study population as Guan et al.’s (*n* = 1590) [26], but studied mortality (Table 4). They used broad age categories (<65, 65–74, and ≥75 years) for their analysis. After adjusting for coronary heart disease, cardiovascular disease, and for several biomarkers, there was an increased risk of mortality for older patients (≥75 years HR 7.86, 95% CI 2.44–25.35; 65–74 years HR 3.43, 95% CI 1.24–9.5). It was not included in the meta-analysis because no other study used comparable age categories.

Zhou et al. 2020 [18] investigated death in 191 COVID-19 patients from two hospitals which were included as part of Chen R et al. 2020 [23] and Guan’s study population and in similar timeframes. Several of the risk factors of interest were missing from the analysis (diabetes, weakened immunity, and respiratory disease). After the adjustment for confounders, an increased risk of death was found for each year of age (OR = 1.10; 95% 1.03–1.17). Since “per year of age” analyses were done, the Zhou et al. study was included in the risk of bias and meta-analysis for death as an outcome. We evaluated the study as having a high risk of bias because not all age-related risk factors were considered, and because biomarkers which were already reflective of COVID-19 disease severity were used for the risk analysis.

Wang D. et al. 2020 [19] studied 107 patients hospitalized at Zhongnan Hospital of Wuhan University and at Xischui Hospital. After adjusting for sex, hypertension, cardiovascular disease, and creatinine concentration, each increased year of age resulted in an 11% increased risk of mortality (OR = 1.11; 95% CI 1.042–1.184). Because not all important age-related risk factors were included in the analysis, and because they adjusted for biomarkers which were reflective of disease severity/COVID-19 infection, the study was rated as having a high risk of bias. Wang D. et al. 2020 [19] was included in our meta-analysis.

In another retrospective study, Wang K. et al. 2020 [20] studied 305 patients hospitalized in First People’s Hospital of the Jiangxia District in Wuhan from 7 January to 11 February 2020. After adjustment for hypertension and fever, there was a 9% increased mortality for every year of age (OR = 1.09; 95% CI 1.054–1.14). Due to the lack of age-related risk factors included in the analysis and the adjustment for fever, which is reflective of COVID-19 infection/disease severity, this study was determined to have a high risk of bias. This study was included in our meta-analysis.

Shi et al. 2020 [21] studied the patients admitted to Renmin Hospital of Wuhan University from 1 January to 23 February 2020, using a retrospective cohort design. They used two models: model 1 considered sex, hypertension, diabetes, coronary heart disease, chronic renal disease, cerebrovascular disease and several biomarkers as categorical variables. Model 2 used sex and the same chronic diseases as model 1, but with the biomarkers as continuous variables. When Model 1 was used, there was a 1% increased risk of death per year of life (OR = 1.01; 95% CI 0.98–1.05) which was borderline statistically significant. Model 2 resulted in a 4% increased risk of death per year of life (OR = 1.04; 95% CI 1.00–1.07), also borderline statistically significant. We evaluated this study as having a high risk of bias, because not every important age-related risk factor was considered and because it used variables which may reflect COVID-19 disease severity. Shi et al. 2020 [21] was included in our meta-analysis.

Sun et al. 2020 [22] identified 244 patients over the age of 60 years of the Sino-French New City Branch of Tongji hospital between 29 January to 5 March 2020. Only patients who were 60 years or older were enrolled. After adjustment for sex, hypertension, previous respiratory diseases and other confounders, there was a 12% increase risk of death per year of life was observed (OR = 1.12; 95% CI 1.01–1.25). This study received a high risk of bias rating because only patients older than 60 years of age were considered, because of missing risk factors, and because biomarkers were used that might indicate a COVID-19 infection/disease severity. Sun et al. 2020 [22] was included in our meta-analysis.

#### 3.3.2. Risk of Bias

The reasons for the exclusion of any study from our meta-analysis/risk of bias assessment for death are summarized in Table 4. In summary, five studies (Zhou et al. 2020 [15], Wang D et al. 2020 [19], Wang K et al. 2020 [20], Shi et al. 2020 [21], and Sun et al. 2020 [22]) were included in the risk of bias assessment and meta-analysis. All the studies included in the meta-analysis for mortality were rated as having a high risk of bias because not all age-dependent risk factors were considered and because the variables used in the analysis may have already indicated COVID-19 disease severity. In addition, Sun et al. 2020 [22] only investigated people over the age of 60, and therefore it received a high-risk in the recruitment procedure domain.

#### 3.3.3. Meta-Analysis and Meta-Regression

**Figure 4:**
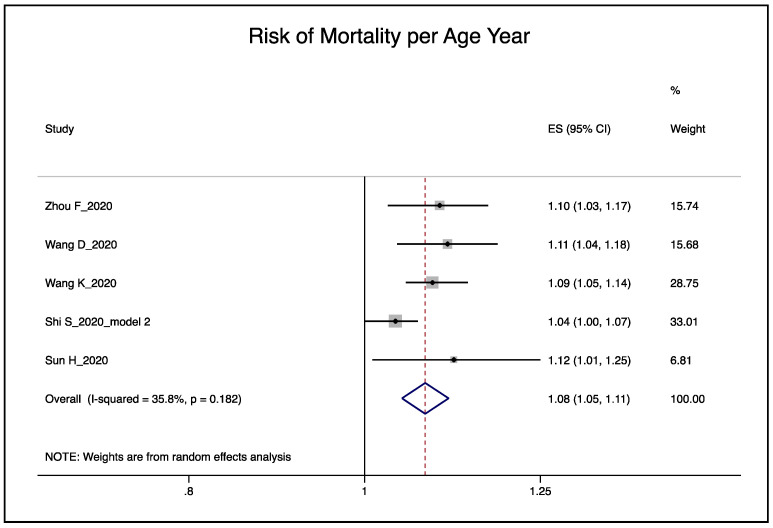
Pooled effect of the risk of age on mortality, random-effects meta-analysis.

**Figure 5:**
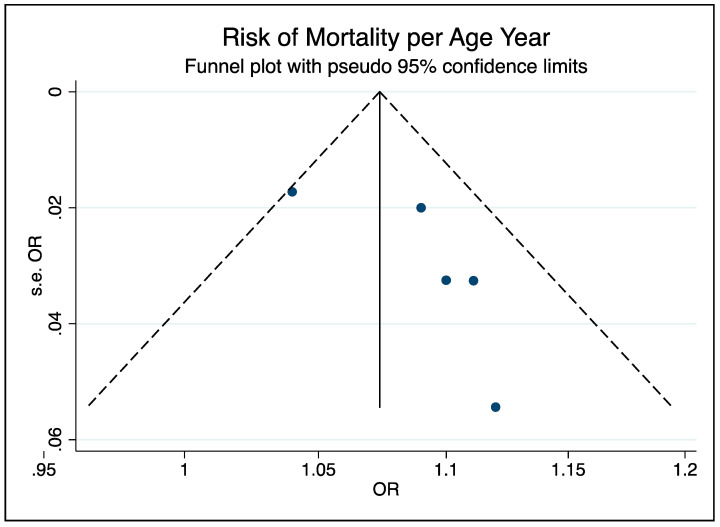
Funnel plot of mortality per age year.

The pooled effect of the five studies [18,19,20,21,22] indicates an 8% increase in the risk of death per year of age (95% CI 3–13%) when Shi et al.’s [21] model with categorized values for the biomarkers was used (Figure 4). The effect remains similar with slightly narrower confidence intervals when model 2 was used with continuous values (RR = 1.08; 95% CI 1.06–1.11). The corresponding funnel plot indicates no evidence of publication bias (Egger’s test *p* = 0.21), see Figure 5 for funnel plot).

The meta-regression from five studies resulted in an intercept of 1.134 (95% CI 1.110–1.158) and a slope of β = 0.978 (95% CI 0.967–0.989). Table 6 shows the estimates of the relative risk by the number of important risk factors adjusted for. The unadjusted age effect resulted in a 13.4% increase in mortality per age year. The maximum number of risk factors used by the studies was five, which would result in a 1.4% increase in death per age year.

The sensitivity analysis shows a statistically non-significant decrease in the relative risk (0.979; 95% CI 0.923–1.039) with over-adjustment; meaning including variables which reflect a current infection (probably due to COVID-19) in the regression model will decrease the calculated relative risk by a factor of 0.979 (Table 7).

### 3.4. Admission to ICU

#### Description of Studies

Chen J. et al. 2020 [16] studied 249 patients at Shanghai Public Health Clinical Center between 20 January and 25 February 2020 (Table 5). After adjusting for sex, cardiovascular and cerebrovascular diseases, endocrine system diseases, digestive system diseases, respiratory system diseases, hepatitis B, malignant tumor, and several biomarkers, there was an increased, borderline statistically significant risk per year of age on the admission to the ICU (OR = 1.06; 95% CI 1.00–1.12).

Because the study [16] did not adjust for all the important risk factors and because it used biomarkers that may be indicative of a COVID-19 infection or COVID-19 severe disease, it was assessed as having a high risk of bias. No meta-analysis could be done for this outcome due to the lack of studies.

### 3.5. Effect of Age-Dependent Risk Factors Adjustment on Age-Related Risk Estimates

According to the meta-regression of five studies, the effect estimate of age on mortality risk decreases with an increasing adjustment for important age-related risk factors. A maximum of five age-dependent risk factors were adjusted for in the included studies. After the adjustment for these five risk factors, the risk per age year decreases from 13.4% (unadjusted value) to 1.4% per year. This translates to about a 20% higher risk of death for a 60 year-old person compared to that of a 50 year-old person due to the (almost) isolated effect of age. Nonetheless, no studies adjusted for all six important risk factors, which would further decrease the age-related risk. 

**Figure 6:**
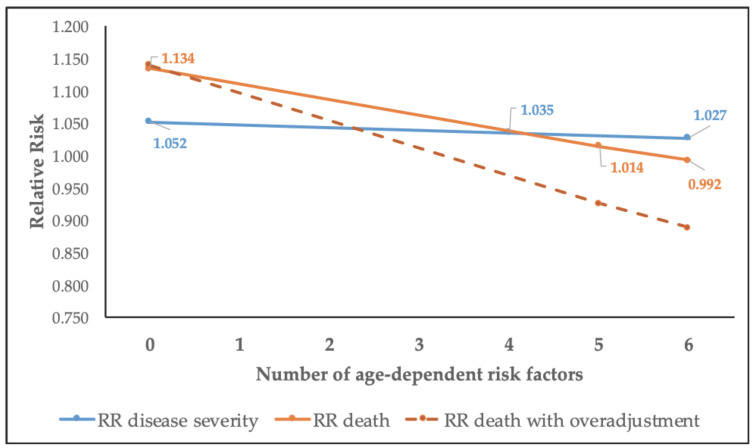
Effect of the adjustment scenarios on the relative risk (RR) of the association between age and COVID-19 disease severity and death.

To illustrate our point more clearly, Figure 6 shows how the effect estimate of disease severity and death decreases with the inclusion of the important age-dependent risk factors. Looking at only disease severity (blue line) and death (orange line), the following is shown: the age-related risk decreases with adjustment for each age-related risk factor (i.e., age-related comorbidities). If we were to extrapolate to the scenario where all six age-dependent risk factors were adjusted for, there would be a 3.2% increased risk per age year for disease severity, and almost no age-related risk for death.

Figure 6 also depicts the effect of over-adjustment in the model, when the models use variables (usually biomarkers) that are already indicative of infection. In this case, the effect estimate is even lower than when just adjusting for the important age-dependent risk factors. Such a scenario is also undesirable, since it leads to a considerable underestimation of the effect.

## 4. Discussion

Our results show an increased age-related risk of COVID-19 disease severity, admission to ICU, and death. However, our risk of bias analysis show that these pooled results are biased: not one study adjusted for all the necessary age-dependent risk factors to obtain the isolated effect of age on COVID-19 disease severity. Further analysis attempting to correct for this bias shows that if important age-related risk factors are taken into account, there is a 2.7% increased risk per age year for disease severity (based on two studies), and almost no age-related risk for death (based on five studies). It appears that age-related comorbidities have a more important weight than age itself.

### 4.1. Strengths and Limitations

To our knowledge, this is the first review to investigate the isolated age-associated risk for a COVID-19 disease severity or death. The main strengths of our research methods were the systematic literature search, and the independent appraisal of titles, abstracts, and full text by two scientists. Our formal risk of bias assessment for the included studies was integrated into the meta-analysis and interpretation of our results. We only included the studies published in peer-reviewed journals, although we included the studies available as a pre-print only due to the time criticality of the research question on hand. There was no indication of publication bias for the risk of severe outcomes or death.

In our meta-regression, we assumed that the effect of each risk factor on the risk reduction was the same and that no interactions were present, which might not be the case. However, it was our wish to illustrate the general consequence of not adjusting for all (known) age-dependent risk factors when estimating the effect of age on COVID-19 disease severity or death. Future studies need to apply models that appropriately incorporate all relevant risk factors.

### 4.2. Methodological Quality of the Included Studies

In our risk of bias analysis, we assessed all the included studies to have biased results, mostly because none included all the important age-related risk factors necessary to estimate the isolated effect of age on disease severity. We therefore stress that future studies control for all of these factors in their analysis. The most common reason for study exclusion was that only the unadjusted risk of age was reported—meaning that only univariate analysis or age—stratified results were reported. The data might be indeed available already—but it has just not been adequately analyzed and published. 

In addition, studies might also have used different definitions of certain conditions, such as hypertension, or the collective terms such as “cardiovascular disease”, or “renal disease”. Furthermore, most studies used biomarkers or disease markers reflective of an infection such as COVID-19 in their analysis. Our sensitivity analysis indicated that the use of those markers would lead to a (statistically not significant) decrease in the relative risk, underestimating the real age-related risk of death. Further studies should restrain from including such variables in their models when studying COVID-19 disease severity to avoid over-adjustment. 

All studies used hospitalized patients as the study population, which may not be reflective of the general population. Therefore, the results of this study refer to the risk of COVID-19 disease severity in hospitalized patients. In order to study the risk of disease severity in COVID-19 infection, it is necessary to study the general population infected with COVID-19 and to determine their outcomes with a prospective cohort study.

### 4.3. Implications for Public Policy

It is necessary to accurately define and target risk groups for COVID-19 disease severity for any prevention measures considered. Taking the example of one of the included studies investigating disease severity [26], the results show that the unadjusted risk of a 50 year old person is compared to that of a 60 year old person, the risk increase is comparable to the risk observed for diabetes, smoking or hypertension (OR per 10 yrs. = 1.65). However, when a more isolated age effect is calculated by adjusting for other important age-related risk factors, the risk due to age decreases by almost a third, this time being lower than the risk caused by diabetes, smoking, hypertension, malignancy or COPD. 

This way, one could make individualized risk profiles to set more transparent and logical recommendations in the case of a lockdown. One could for instance compare the risk of a 45 year-old person suffering from diabetes to the risk of a 60 year old person with no underlying illnesses and come to the conclusion that the younger person with diabetes would have a higher risk for COVID-19 disease severity. Thus, it is particularly important at this time that the general population is aware of their underlying conditions and to continue to attend health screenings and medical check-ups. Policy makers should in turn promote screenings at this time.

Overall, it seems arbitrary to target persons over the age of 60 years as a high-risk group, solely based on age. There are estimates that up to 20–30% of people between the age of 60 and 65 have no underlying chronic disorders [27]. Even if this estimate were lower, targeting solely by age group would bring potentially unnecessary and unjustified consequences. This undifferentiated classification may encourage the discrimination of older people in society at large, which has already been reported during the COVID-19 pandemic [28]. Serious negative repercussions can result for older people, including biographical constraints, psychological problems, and economic hardship [28,29,30]. In terms of the older worker, such undifferentiated classification would also be difficult to reconcile with the declaration of the Council of the European Union of July 2012, which, under the heading “Prevention of age discrimination” [31], cites “refraining from using age as a decisive criterion for assessing whether a worker is fit for a certain job or not”. Since age is an essential and inevitable characteristic of a person, particular caution seems necessary when defining age-specific exclusion criteria for certain jobs. In this respect, it should be pointed out that the social isolation often associated with the selective absence of older employees from the workplace can in principle lead to depressive and post-traumatic stress symptoms [32].

## 5. Conclusions

The indication of a rather weak influence of age on COVID-19 disease severity and death after adjustment for important age-dependent risk factors should be taken in consideration when implementing age-related preventative measures. 

## Figures and Tables

**Table 1:** Study eligibility criteria.

	Inclusion Criteria	Exclusion Criteria
Population	General population infected with COVID-19 (both sexes, all ages)	All others
Intervention(s), exposure(s)	Age, in years	All other exposures which do not include age
Comparator/control	Persons from the same study population as the cases differing in age	Other populations which are not comparable to the cases in age
Outcomes	Disease severity due to infection with COVID-19: risk of hospitalization, admission to intensive care unit (ICU), intubation, death, other markers of severe disease due to COVID-19 Risks measured as adjusted hazard ratios, risk ratios, odds ratios	Other outcomes
Study design	cross-sectional, case-control, and cohort studies	Randomized controlled trials (RCTs), qualitative studies, ecological studies, case reports, experiments, comments, letters, editorials, congress abstracts, posters

**Table 2:** Characteristics of the included studies.

Author, Year Country [Ref]	Study Design	Population Sampling	Age/Sex	Time Period of Study	Age Categories Used	Outcome Measurement
Guan, W. * 2020 China [26]	Retrospective cohort	COVID-19 laboratory-confirmed hospitalized patients (13.5% of cases as of 1/31/2020) from 575 hospitals (32% of all certified hospitals for COVID-19) and 31 areas across mainland China Complete random sampling could not be done *n* = 1590	Mean age: 48.9 yrs. Male: 904 (57.3%) Female: 674 (42.7%)	12/11/2019–01/31/2020	Per year and <65 yrs. vs. ≥65 yrs.	Composite measure of admission to intensive care unit (ICU), invasive ventilation, or death
Chen, R. * 2020 China [23]	Retrospective cohort	Same population as above (*n* = 1590)	Same as above	Unknown–01/31/2020	65–74 yrs. vs. <65 yrs. and ≥75 yrs. vs. <65 yrs.	Death
Liang, W. * 2020 China [17]	Retrospective cohort	Same population as above (*n* = 1590)	Same as above	11/21/2019–01/31/2020	Per year	Composite measure of admission to intensive care unit (ICU), invasive ventilation, or death
Du, R.-H. * 2020 China [24]	Prospective cohort	Patients hospitalized at Wuhan Pulmonary Hospital, Wuhan City (most likely a sub-set of Guan et al. 2020′s population) All patients included *n* = 179	Mean age: 57.6 yrs. SD: 13.7 yrs. Male: 97 (54.2%) Female: 82 (45.8%)	12/25/2019–02/07/2020	≥65 yrs. vs. <65 yrs.	Death
Zhou, F. * 2020 China [18]	Retrospective cohort	Two cohorts of adult patients (≥18 yrs.) from Jinyintan Hospital and Wuhan Pulmonary Hospital (Wihan, China). *n* = 191 Jinyintan Hospital: *n* = 135 Wuhan Pulmonary Hospital: *n* = 56	Median age: 56.0 yrs. IQR: 46.0–67.0 yrs. Male: 119 (62%) Female: 72 (38%)	12/29/2019–01/31/2020	Per year	Death
Wang, D. 2020 China [19]	Retrospective cohort	Patients hospitalized at Zhongnan Hospital of Wuhan University and Xishui Hospital, Hubei Province *n* = 107 patients	Median age: 51 yrs. Range: 19–92 yrs. Male: 57 (53.3%) Female: 50 (46.7%)	unknown–02/10/2020	Per year	Death
Wang, K. 2020 China [20]	Retrospective cohort	Participants diagnosed with COVID-19 and hospitalized in First People’s Hospital of Jiangxia District in Wuhan *n* = 305	Mean age: 47.8 yrs. SD: 15.1 yrs. Male: 142 (46.6%) Female: 163 (53.4%)	01/07/2020–02/11/2020	Per year	Death
Chen, J. 2020 China [16]	Retrospective cohort	Patients at Shanghai Public Health Clinical Center (SPHCC). *n* = 249	Median age: 51 yrs. IQR: 36–64 yrs. Male: 126 (50.6%) Female: 123 (49.4%)	01/20/2020–02/25/2020	per year	Admission to ICU
Chen, C. ** 2020 China [15]	Retrospective cohort	Patients admitted to fever ward in Tongji Hospital, Tongji Medical College in Huazhong University of Science and Technology *n* = 150	Median age: Non-critical group: 57.1 ± 15.6 yrs. Critical group: 68.5 ± 13.6 yrs. Male: 84 (56%) Female: 66 (44%)	January to February 2020	per year	Composite measure of critical and severe coronavirus pneumonia (with one of the following conditions): respiratory failure and mechanical ventilation; shock; combined with failure of other organs should be treated in the ICU
Meng, Y. ** 2020 China [25]	Retrospective cohort	Patients hospitalized at Tongji Hospital in Wuhan, China *n* = 168	Mean age: 56.7 yrs. SD: 15.1 yrs. Male: 86 (51.2%) Female: 82 (48.8%)	Hospitalized 01/16/2020–02/04/2020 and monitored up to 03/24/202	0–59 yrs. 60–79 yrs. ≥80 yrs.	Critically ill cases defined as patients who met any of the following criteria: developed respiratory failure requiring intubation; presented with shock; developed other organ failure or were admitted to ICU
Shi S. 2020 China [21]	Retrospective cohort	All consecutive patients admitted to Renmin Hospital of Wuhan University with lab-confirmed COVID-19 *n* = 671	Median age: 63 yrs. IQR: 50–72 yrs. Male: 322 (48.0%) Female: 349 (52.0%)	01/01/2020–02/23/2020	Per year	Death
Sun, H. 2020 China [22]	Retrospective cohort	Participants identified from inpatients of the Sino-French New City Branch of Tongji hospitals with 1085 beds for treating Covid-19 designated by the government Participants 60 yrs. and older with definitive outcomes by March 5, 2020 were enrolled *n* = 244	Discharged: Median age: 67 yrs. Range: 64–72 yrs. Died: Median age: 72 yrs. Range: 66–78 yrs. Male: 133 (54.5%) Female: 111 (45.5%)	01/29/2020–03/05/2020	Per year	Death

* Studies marked have the same population or a sub-group of the same population; ** Studies marked have the same population or a sub-group of the same population; IQR: interquartile range; yrs.: years; ICU: intensive care unit.

**Table 3:** Characteristics and results of included studies using composite measures of severe outcomes.

Author, Year [Ref]	Confounders/ Age-Dependent Risk Factors Used in the Model In RoB/Meta-Analysis (Yes/No) Number of Age-Dependent Risk Factors for Meta-Regression	Type of Analysis Number of Cases/Number of Non-Cases	Results
Guan, W.* 2020 [26]	Malignancy, COPD, diabetes, hypertension, smoking Comorbidity, defined as: hypertension, other cardiovascular disease, cerebrovascular diseases, diabetes, hepatitis B infections, COPD, malignancy, immune deficiency In meta-analysis: yes Number of age-dependent risk factors for meta-regression: 4 (malignancy, COPD, diabetes, hypertension)	Cox proportional hazards regression cases = 131 (8.3%): died: *n* = 50 ICU: *n* = 99 Invasive ventilation: *n* = 50 Non-cases: 1459 (91.7%)	Association of age (per yr.) and severe outcome: Unadjusted OR: 1.051 95% CI (1.039−1.064) OR adjusted for malignancy: 1.050, 95% CI (1.037−1.062) OR adj. for malignancy, COPD: 1.045, 95% CI (1.032−2.058) OR adj. for malignancy, COPD, diabetes: 1.041, 95% CI (1.027−1.055) OR adj. for malignancy, COPD, diabetes, hypertension: 1.036, 95% CI (1.022−1.051) OR adj. for malignancy, COPD, diabetes, hypertension, smoking: 1.036, 95% CI (1.022−1.050 Number of comorbidities and severe outcome by age: 1 comorbidity: <65 yrs.: HR 2.210, 95% CI (1.234−3.960) ≥65 yrs.: HR 1.801, 95% CI (0.912−3.554) ≥2 comorbidities: 65 yrs.: HR 3.332, 95% CI (1.557−7.132) ≥65 yrs.: HR 2.724, 95% CI (1.409−5.265) Smoking (yes vs. no) 65 yrs.: HR 1.495, 95% CI (0.641−3.488) ≥65 yrs.: HR 1.534, 95% CI (0.813−2.892)
Liang, W.* 2020 [17]	X-ray abnormality, hemoptysis, dyspnea unconsciousness, number of comorbidities, cancer history, neutrophil to lymphocyte ratio, lactate dehydrogenase, direct bilirubin In meta-analysis: no (same population as Guan et al. 2020 [26])	Logistic regression cases = 131 (8.3%) Non-cases = 1459 (91.7%)	Association of age (per yr.) and severe outcome: Adj. OR: 1.03, 95% CI (1.01−1.05)
Chen, C.** 2020 [15]	Sex, increased NT-proBNP, increased cTnI, increased hs_CRP, increased blood creatinine, hypertension, diabetes, history of previous coronary heart disease In meta-analysis: yes Number of age-dependent risk factors for meta-regression: 4 (NT-proBNP increased, hypertension, diabetes, history of previous coronary heart disease)	Logistic regression cases = 24 Non-cases = 126	Association of age (per yr.) and severe coronavirus pneumonia: Unadj. OR: 1.056, 95% CI (1.020−1.092) Adj. OR: 1.019, 95% CI (0.963−1.077)
Meng, Y.** 2020 [25]	Comorbidities: hypertension, diabetes, cardiovascular disease, chronic kidney disease, cerebrovascular disease, COPD, malignancy In meta-analysis: no (same or sub-population as Chen et al. 2020 [15])	Logistic regression Cases = Died: 17 (8.9%) Critically ill: 48 (28.6%) Non-cases = 136 (81%)	Association of age and severe outcome: Women and men Age 0−59 yrs.: Ref. Age 60−79 yrs.: Unadj. OR: 5 (2−10) ^†^ Adj. OR: 3 (0.9−8) ^†^ ≥80 yrs.: Unadj. OR: 10.968 (3.005−40.037) Adj. OR: 10 (2−40) ^†^ Men: Age 0−59 yrs.: Ref ≥80 yrs.: Unadj. OR: 10 (1−50) ^†^ Adj. OR: 9.333 (1.618−53.845) Women: Age 0−59 yrs.: Ref ≥80 yrs.: Unadj. OR: 20 (2−200) ^†^ Adj. OR: 10.161 (0.911−113.346)

* Studies have the same population or a sub-group of the same population; ** Studies have the same population or a sub-group of the same population; HR: hazards ratio; OR: odds ratio; yrs.: years; RoB: risk of bias assessment; Adj.: adjusted; Unadj.: unadjusted; COPD: chronic obstructive pulmonary disease; NT-proBNP: N terminal prohormone of brain natriuretic peptide; cTnI: cardiac troponin I; hs CRP: high-sensitivity C-reactive protein. ^†^ estimated from Figure 3 from Meng Y. et al. 2020 [25].

**Table 4:** Characteristics and results of included studies using death as outcome.

Author, Year [Ref]	Confounders/ Age-Dependent Risk Factors Used in Model In RoB/Meta-Analysis (Yes/No) Number of Age-Dependent Risk Factors for Meta-Regression	Type of Analysis Number of Cases/Number of Non-Cases	Results
Zhou, F. * 2020 [18]	Coronary heart disease, Sequential Organ Failure Assessment (SOFA) score, lymphocite count, D-dimer In meta-analysis: yes Number of age-dependent risk factors for meta-regression: 1 (coronary heart disease)	Logistic regression Only significant factors from univariate taken in multivariate model Cases = 54 (28.3%) Non-cases = 137 (71.7%)	Association between age (per yr.) and in-hospital mortality: Unadj. OR: 1.14, 95% CI (1.09−1.18) Adj. OR: 1.10, 95% CI (1.03−1.17))
Du, R.H. * 2020 [24]	Cardiovascular or cerebrovascular diseases, CD3 + CD8+ T cells ≤ 75 cell/ug, Cardiac troponin I ≥ 0.05 ng/mL In meta-analysis: no (same or sub-population as Zhou et al. 2020 [18] and larger age categories used in analysis)	Logistic regression Cases = 21 Non-cases = 158	Association between age and mortality: Unadj. OR 0−49 yrs.: Ref. 50−64 yrs.: 2.673, 95% CI (0.859−8.318) ≥65 yrs.: 9.740, 95% CI (3.113−30.476) Adj. OR < 65 yrs.: Ref ≥65 yrs.: 3.765 (1.146–17.394)
Chen, R. * 2020 [23]	Coronary heart disease (CHD), cardiovascular disease (CVD), dyspnea, PCT > 0.5 ng/mL, AST > 40U/L, TBIL, Cr In meta-analysis: no (same or sub-population as Zhou et al. 2020 [18] and large age categories used in analysis)	Cox regression Cases = 50	Association between age and mortality: Age ≥ 75 yrs. vs. <65 yrs.: Adj. HR: 7.86 (95% CI: 2.44–25.35) Age 65–74 yrs. vs. <65 yrs. Adj. HR: 3.43 (95% CI: 1.24–9.5)
Wang, D. 2020 [19]	Sex, hypertension, cardiovascular disease, creatinine concentration In meta-analysis: yes Number of age-dependent risk factors for meta-regression: 2 (hypertension, cardiovascular disease)	Logistic regression Cases = 19 Non-cases = 88	Association between age (per yr.) and mortality: Unadj. OR: 1.102, 95% CI (1.054−1.152) Adj. OR: 1.111, 95% CI (1.042−1.184)
Wang, K. 2020 [20]	Hypertension, fever In meta-analysis: yes Number of age-dependent risk factors for meta-regression: 1 (hypertension)	Logistic regression Cases = 22 (7.2%) Non-cases = 283 (92.8%)	Association between age (per yr.) and mortality: Adj. OR: 1.09, 95% CI (1.054−1.14)
Shi S. 2020 [21]	**Model 1:** Sex, hypertension, diabetes, coronary heart disease, chronic renal disease, cerebrovascular disease, and following biomarkers as categorical variables: procalcitomin, c-reactive protein, CK-MB, MYO, cTnI, NT-proBNP **Model 1:** Sex, hypertension, diabetes, coronary heart disease, chronic renal disease, cerebrovascular disease, and following biomarkers as continuous variables: procalcitomin, c-reactive protein, CK-MB, MYO, cTnI, NT-proBNP In meta-analysis: yes Number of age-dependent risk factors for meta-regression: 5 (hypertension, diabetes, coronary heart disease/cerebrovascular disease, chronic renal disease, NT-proBNP)	Cox regression Cases = 62 Non-cases = 609	Association between age (per yr.) and in-hospital mortality: Model 1 Adj. OR: 1.01, 95% CI (0.98−1.05) Model 2 Adj. OR: 1.04, 95% CI (1.00−1.07)
Sun, H. 2020 [22]	Sex, SpO2, heart rate, respiratory rate, consciousness disorders, hypertension, previous respiratory diseases, WBC count, LYM count, NT-prBNP, PCT, hs-TnI, D-dimer, ALT, AST, creatinine, eGFR, hs-CRP In meta-analysis: yes Number of age-dependent risk factors for meta-regression: 3 (hypertension, NT-proBNP, hs-Tnl)	Logistic regression Cases = 122 Non-cases = 123	Association between age (per yr.) and mortality: Adj. OR: 1.12, 95% CI (1.01−1.25)

* Studies have the same population or a sub-group of the same population; HR: hazards ratio; OR: odds ratio; yrs.: years; RoB: risk of bias assessment; Adj.: adjusted; Unadj.: unadjusted; PCT: procalcitonin; AST: aspartate transaminase; TBIL: total bilirubin; CK-MB: creatine kinase myocardial band; MYO: myoglobin; cTnI: cardiac troponin I; NT-proBNP: N terminal prohormone of brain natriuretic peptide; LYM: lymphocyte; hs-TnI: high-sensitive troponine I; ALT: alanine transaminase; eGFR: estimated glomerular filtration rate; hs-CRP: high-sensitivity C-reactive protein.

**Table 5:** Characteristics and results of included studies using admission to ICU as outcome.

Author, Year [Ref]	Confounders/ Age-Dependent Risk Factors Used in the Model In RoB/Meta-Analysis (Yes/No) Number of Age-Dependent Risk Factors for Meta-Regression	Type of Analysis Number of Cases/Number of Non-Cases	Results
Chen, J. 2020 [16]	Sex, comorbidity (cardiovascular and cerebrovascular diseases, endocrine system diseases, digestive system diseases, respiratory system diseases, chronic hepatitis B, malignant tumor), white blood cells, lymphocytes, C-reactive protein, albumin, lactate dehydrogenase, estimated glomerular filtration rate, CD4 T cell counts In meta-analysis: no (insufficient studies)	Logistic regression Cases = 22 Non-cases = 227	Association between age (per yr.) and risk of admission to ICU: Unadj. OR: 1.08, 95% CI (1.04−1.13) Adj. OR: 1.06, 95% CI (1.00−1.12)

OR: odds ratio; RoB: risk of bias assessment; ICU: intensive care unit.

**Table 6:** The effect of the number of age-related risk factors included in the multivariate model on the relative risk (RR) estimate for disease severity and death.

Number of Age-Related Risk Factors	RR_age_ Disease Severity ^†^	RR_age_ Death ^‡^
0	1.052	1.134
1	1.047	1.109
2	1.043	1.084
3	1.039	1.060
4	1.035	1.037
5	1.031 *	1.014
6	1.027 *	0.992 *

^†^ Effect not significant (*p* = 0.22); ^‡^ Effect significant (*p* = 0.007); * Effect estimate is an extrapolation.

**Table 7:** Sensitivity analysis: the effect of inclusion of the variables reflective of infection in the multivariate model.

Component	Model Estimate (95% CI)
Intercept	1.139 (1.125, 1.153)
β (risk factor)	0.981 (0.966, 0.998)
β (presence of over-adjustment)	0.979 (0.923, 1.039)
